# Controlled Chemical Synthesis of Color Centers in Nanocrystalline Silicon Carbide

**DOI:** 10.3390/nano16100627

**Published:** 2026-05-19

**Authors:** Sarah Morais Bezerra, Gabor Bortel, Sándor Kollarics, Adam Gali, David Beke

**Affiliations:** 1 HUN-REN Wigner Research Centre for Physics, Institute for Solid State Physics and Optics, H-1121 Budapest, Hungary; 2 Department of Physical Chemistry and Materials Science, Faculty of Chemical Technology and Biotechnology, Budapest University of Technology and Economics, Műegyetem Rakpart 3, H-1111 Budapest, Hungary; 3 SOLEIL Synchrotron, L’Orme des Merisiers, RD128, 91190 Saint Aubin, France; 4 Department of Physics, Institute of Physics, Budapest University of Technology and Economics, Müegyetem Rakpart 3, H-1111 Budapest, Hungary; 5 HUN-REN–BME Condensed Matter Research Group, Budapest University of Technology and Economics, Müegyetem Rakpart 3, H-1111 Budapest, Hungary; 6 Department of Atomic Physics, Institute of Physics, Budapest University of Technology and Economics, Müegyetem Rakpart 3, H-1111 Budapest, Hungary; 7 MTA-WFK Lendület “Momentum” Semiconductor Nanostructures Research Group, H-1525 Budapest, Hungary; 8 Kandó Kálmán Faculty of Electrical Engineering, Óbuda University, Tavaszmező 17, H-1084 Budapest, Hungary

**Keywords:** silicon carbide, color centers, divacancy, qubit, solid state synthesis

## Abstract

Silicon carbide is a promising material for optically and spin-active point defects relevant to quantum applications. Quantum-relevant color centers are commonly generated by irradiation or implantation, which require specialized infrastructure and may introduce collateral lattice damage. Here, we present a chemical approach in which the influence of synthesis temperature, high-energy ball milling, and aluminum addition on formation, polytype distribution, and defect formation in SiC is investigated. We found that it is possible to create quantum-relevant defects throughout the chemical synthesis, and the temperature and mechanical activation are the dominant parameters governing defect generation. Photoluminescence and electron paramagnetic resonance spectroscopy demonstrate that low synthesis temperatures (1050–1150 °C) in high-energy ball-milled samples yield silicon vacancy and divacancy-related color centers, evidenced by characteristic near-infrared PL emission and high-spin EPR signals with zero-field splitting values D ≈ 1.3 GHz and D ≈ 270 MHz, consistent with neutral divacancies and V_Si_–C_Si_ complex centers, respectively. An additional EPR signal at D ≈ 650–780 MHz, not matched by any previously reported defect configuration in SiC, is tentatively assigned to a second-nearest-neighbor divacancy-like (V_Si_–V_C_) pair.

## 1. Introduction

Silicon carbide is a wide-bandgap semiconductor that has emerged as a key material for next-generation electronic [1], photonic [2], and quantum technologies [3] due to its exceptional thermal stability and chemical robustness. In recent years, optically and spin-active point defects in SiC, most notably silicon vacancies (V_Si_^−^) [4] and divacancies (VV) [5], have attracted intense interest as solid-state spin qubits and near-infrared single-photon emitters. These color centers are often compared to the negatively charged nitrogen-vacancy (NV^−^) center in diamond in terms of spin coherence times reaching milliseconds at room temperature, while offering complementary advantages, i.e., near-infrared optical addressability (850–950 nm for V_Si_^−^ and ~1100 nm for VV), CMOS-compatible host processing, natural isotopic abundance favorable for spin coherence, and the ability to engineer defect properties through polytype selection. Critically, SiC’s compatibility with industrial-scale fabrication and its substantially lower cost relative to diamond positions it as the most technologically accessible platform for scalable quantum sensing, spin-based magnetometry, and quantum network nodes [6,7].

A central challenge for SiC-based quantum technologies lies in the controlled generation and stabilization of specific defect centers while maintaining suitable crystal quality and polytype composition [8]. The electronic structure, optical emission, and spin properties of V_Si_^−^ and VV defects depend strongly on the local lattice environment, including polytype, crystallographic site, and defect charge state [9].

Intrinsic color centers in SiC are typically generated through vacancy creation induced by high-energy particles [6,10], including electrons [11,12], ions [13], neutrons [14], protons [15], and focused Si ion beams [10]. Electron and neutron irradiation generally produce defects without spatial selectivity, whereas ion- and proton-based approaches can provide a degree of localization because the defect distribution is governed by the stopping range of the incoming particles in the crystal. This enables the generation of defects at defined depths and, to some extent, at controlled concentrations, which can subsequently be characterized by electron paramagnetic resonance (EPR) and photoluminescence (PL) spectroscopy [8]. However, irradiation-based routes suffer from fundamental limitations: they introduce a broad spectrum of unintended defect species and extended lattice damage alongside the target centers, require dedicated particle accelerator or reactor facilities that are costly and non-scalable, and provide no direct control over the resulting defect charge states. Synthetic strategies that simultaneously govern polytype distribution, crystallite size, and defect concentration through bench-top chemistry are therefore highly desirable for the rational and scalable engineering of SiC quantum emitters, especially in applications where nanostuructured emitters are favored. Nevertheless, the direct chemical synthesis of optically active, spin-coherent point defects in nanocrystalline SiC remains insufficiently established [16,17].

Conventional SiC synthesis, such as the Acheson process [18], often relies on oxygen-containing precursors such as SiO_2_, where carbothermal reduction produces CO-based gaseous species that facilitate phase formation [19] and are optimized to minimize defect concentrations rather than to engineer specific vacancy populations. These conventional approaches therefore provide little control over defect type, charge state, or concentration and are fundamentally ill-suited for the deliberate synthesis of vacancy-rich materials. Oxygen-free synthesis routes using fluorinated carbon sources, such as poly(tetrafluoroethylene) (PTFE), offer an alternative pathway in which volatile fluoride species govern reaction thermodynamics, modify elemental ratios, and directly influence vacancy formation mechanisms [20,21,22].

In these synthesis methods, high-energy ball milling is a common process to reduce precursor size, increase homogeneity, and decrease particle size after the reaction [18]. Most commonly, HEBM is used as an additional degree of control by mixing and even mechanically activating solid-state reactions, thereby increasing defect density and enhancing diffusion during synthesis. For SiC, when combined with reactive precursors [23], HEBM can significantly influence polytype formation, crystallite size, and defect stabilization, as observed by Yang et al. [21]. The introduction of metallic additives, such as aluminum [24], further modifies both the mechanical and chemical aspects of the synthesis. As a ductile material, Al can also act as an activator by altering milling dynamics and through reactions with Si and C, lowering reaction temperatures and altering polytype formation [17]. Understanding how these parameters influence the formation and charge state of quantum-relevant defects, however, remains an open and important question.

In our previous studies, we showed that HEBM and Al additions in specific amounts reduced crystallite size and lowered the reaction temperature [17,25]. It has been shown that a low amount of Al (5 mol%) improved the material properties, while a higher amount increased the formation of optically active defects in SiC. However, no divacancy-related centers were found under those conditions. The present study systematically varies temperature, HEBM time, and Al content and reports the detection of neutral-divacancy and modified silicon-vacancy EPR signatures in this chemically synthesized system.

## 2. Materials and Methods

Activated carbon (C, NORIT^®^ A SUPRA EUR USP, Sigma-Aldrich, Merck KGaA, Darmstadt, Germany), silicon (Si) powder (~325 mesh, 99% trace metals basis, Sigma-Aldrich, Merck KGaA, Darmstadt, Germany), aluminum powder (Al, ≥91% complexometric) and poly(tetrafluoroethylene) powder (PTFE, 1 µm particle size) were mixed in an HEBM apparatus (Planetary Micro Mill, PULVERISETTE 7, FRITSCH, Fritsch GmbH, Idar-Oberstein, Germany) with either 0% or 10 mol% of Al at 500 RPM for 3 h with a 1:10 Ball to Powder (BPR) ratio. The samples without milling (hereafter, 0 h) were mixed in a homemade mixer at 75 RPM overnight to ensure thorough mixing without milling. Isopropanol was used as a medium to facilitate mixing and milling and was added until the samples had an oil-like consistency. During milling, the samples were kept under argon to prevent oxidation.

After this process, samples were dried and pressed into pellets using a few drops of 5 *w*/*w*% polyvinyl alcohol (PVA) solution to maintain their integrity. Then, the samples were transferred into a Carbolite (CTF 18/–/300) Tube Furnace, Carbolite Gero Ltd., Hope, UK) with varying reaction temperatures (1050, 1150, 1250, 1350, and 1450 °C), with a temperature ramp of 5 °C per minute, also under argon atmosphere. During the ramp, all samples were initially subjected to a dwell at 200 °C for 60 min to ensure that no water remained during the reaction. Afterward, the samples were heated to the desired temperature and held at that temperature for another 60 min. Then the samples were cooled overnight and cleaned.

The cleaning process began with the removal of unreacted carbon by heating the samples in air at 650 °C for 10 h. After that, the samples were loaded into a Teflon beaker; a prepared solution of HF, HNO_3_, and H_2_O (1:1:10) was added; and the mixture was gently stirred. The mixture was left overnight to remove Al and unreacted Si. After this, the samples were washed until the pH reached 5, then dried and prepared for measurements.

The reaction yield was calculated by measuring the weights of the precursors and the cleaned product and, when necessary, corrected based on the composition analysis results from powder X-ray diffraction measurements.

The measurements performed were Powder X-ray diffraction (PXRD), Raman microscopy, Photoluminescence Spectroscopy (PL), and Electron Paramagnetic Resonance (EPR or ESR) to first characterize the samples and then assess whether photoluminescent and paramagnetic properties were achieved.

PXRD measurements were performed using a Huber G670 Guinier Imaging Plate Camera (HUBER Diffraktionstechnik GmbH & Co. KG, Rimsting, Germany) with Cu Kα_1_ radiation (1.5406 Å). The samples were measured in special glass capillaries (Mark-tubes, WJM-Glas Müller GmbH, Berlin, Germany) with a diameter of 0.5 mm and a wall thickness of 0.01 mm to minimize background interference. Rietveld refinement was performed using Topas Academic to characterize the polytype formation and crystallite size.

PL measurements were performed at room temperature (~295 K) in a Renishaw inVia confocal Raman microscope (Renishaw plc, New Mills, England) equipped with a 532 nm excitation laser and 1800 lines per mm gratings. Near-infrared photoluminescence measurements were performed at room temperature by using 980 nm laser excitation (RLTMDL-980-2W-3, 980 nm, (Roithner GmbH, Vienna, Austria), 0.5 W output power, 1 cm^2^ illuminated area) and photon detection with an infrared spectrometer (NearQuest, Ocean Optics, Inc., Orlando, FL, USA).

EPR measurements were performed at room temperature (~295 K) using a Magnettech MiniScope (MS-400 X-band, Magnettech GmbH, Berlin, Germany) spectrometer equipped with a TE_102_ rectangular resonator. The magnetic field was swept from 286 to 386 mT (9.38 GHz, 0.1 mT modulation amplitude, and 10 mW microwave power).

## 3. Results and Discussion

**Figure 1:**
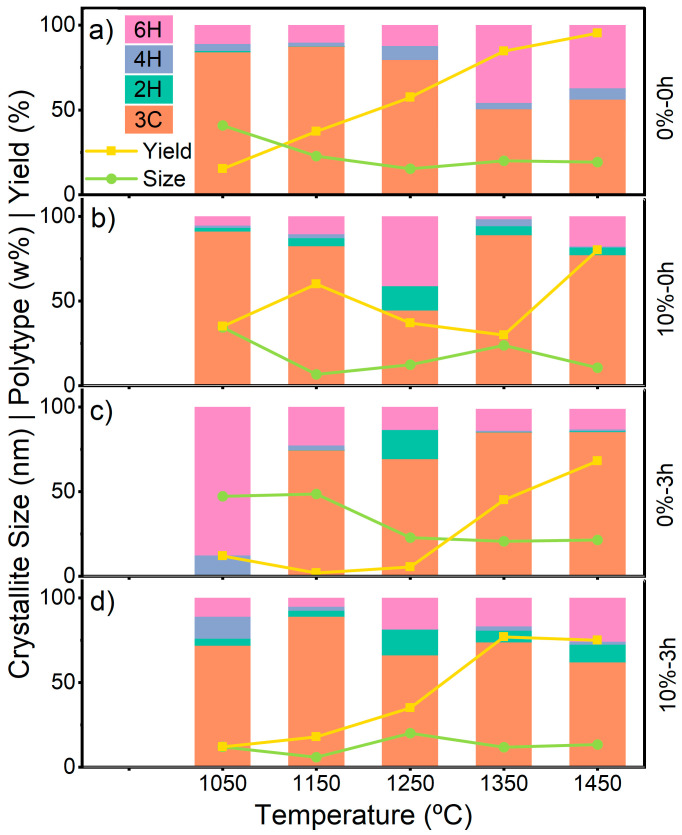
PXRD results from the different samples made (**a**) 0%-0 h, (**b**) 10%-0 h, (**c**) 0%-3 h, and (**d**) 10%-3 h. The percentage is related to the amount of Al added in mol, and the hour mark is related to the HEBM processing time.

The powder X-ray diffraction provided the primary structural characterization of the synthesized samples (Figure 1). The diffraction data were systematically organized and analyzed to allow accurate comparison across processing conditions. Attention was given to polytype distribution, yield, crystallite size, and defect formation as a function of temperature, aluminum addition, and HEBM.

The PXRD diffractograms (Figure S1) indicate the presence of SiC, Si, and Al_2_O_3_ prior to cleaning. For some samples, Si was the main phase because the reaction yield was too low. Stacking-fault-related diffraction features—visible as an asymmetric peak in the 33–35° 2θ region (close to the 4H 101/6H 102/2H 100 reflections), indicated by an arrow in Figure S1—were observed only in samples that contained Al during synthesis.

Regarding polytype concentration, multiple SiC polytypes were identified, consistent with the known polytypism of SiC. In non-milled, Al-free samples, increasing temperature led to higher concentrations of hexagonal polytypes, whereas milled, Al-free samples synthesized at the same temperatures exhibited a marked increase in 3C-SiC content, except for the sample made at 1050 °C, which did not contain 3C-SiC. This observation is quite surprising, as most reports on powder synthesis, including the reaction between elemental Si and PTFE [26], identify 3C-SiC as the dominant phase. Indeed, 3C-SiC is the low-temperature, rapid formation polytype typically obtained in many powder syntheses. Additionally, Al addition consistently promoted the formation of the 2H-SiC polytype. For samples containing Al and subjected to HEBM, no significant variation in cubic concentration was observed.

Both high-energy ball milling and the addition of Al to the precursor mixture are generally expected to reduce the reaction temperature required for SiC formation [27]. We considered product yield and crystallite size as indirect indicators of the effective reaction temperature, since low or negligible SiC yield is typically expected below the ignition temperature. Contrary to this expectation, our results indicate that HEBM increased the apparent reaction temperature. In the samples prepared without HEBM, no clear onset of product yield could be identified to determine a distinct reaction temperature threshold, and yields around 50% were achieved at 1150 °C, irrespective of the presence of Al. By contrast, in the HEBM-treated samples, a clear onset of SiC formation appeared at 1250 °C.

The addition of Al led to a slight improvement in yield, which was significant only in the milled samples, whereas the non-milled samples containing Al showed fluctuating yield values. This behavior is likely related to the inhomogeneous distribution and limited dispersibility of Al powder, resulting from insufficient mixing of the precursor mixture.

In the Al-free samples, the crystallite size showed an opposite trend to the product yield. The observation that more than 10% SiC was formed even at the lowest investigated reaction temperature suggests that locally activated regions exist in the Si-C system, enabling SiC formation at or even below 1050 °C. The inverse trend in crystallite size for the Al-free samples may therefore be explained by the presence of such activated regions, where nucleation begins at a lower temperature and is followed by relatively slow crystal growth. As the temperature increases, progressively more sites become activated, increasing the number of nuclei formed and thereby decreasing the average crystallite size [28]. This interpretation is also consistent with the absence of a well-defined reaction onset in the non-milled samples. The presence of Al reduced the crystallite size as reported earlier [17] and eliminated the correlation between reaction yield and crystallite size.

The applied HEBM may have eliminated the active sites while failing to provide effective mechanical activation. Consequently, SiC formation started at the previously reported temperature of 1250 °C.

Nevertheless, sufficient SiC was formed at most applied reaction temperatures to warrant further investigation. In general, higher synthesis temperatures yield less defective material, as indicated by PXRD and Raman results (Figure S2). Since the average crystallite size did not increase with synthesis temperature, the PXRD peaks have similar full-width-at-half-maximum (FWHM); however, both phase and polytype purity improved at higher synthesis temperatures. In the Raman spectra (Figure S2), the SiC TO (~796 cm^−1^) and LO (~972 cm^−1^) modes sharpened, and their FWHM decreased; the disorder-related background between 700 and 950 cm^−1^ diminished. Quantitative Raman parameters extracted from these spectra are summarised in Table S7.

**Figure 2:**
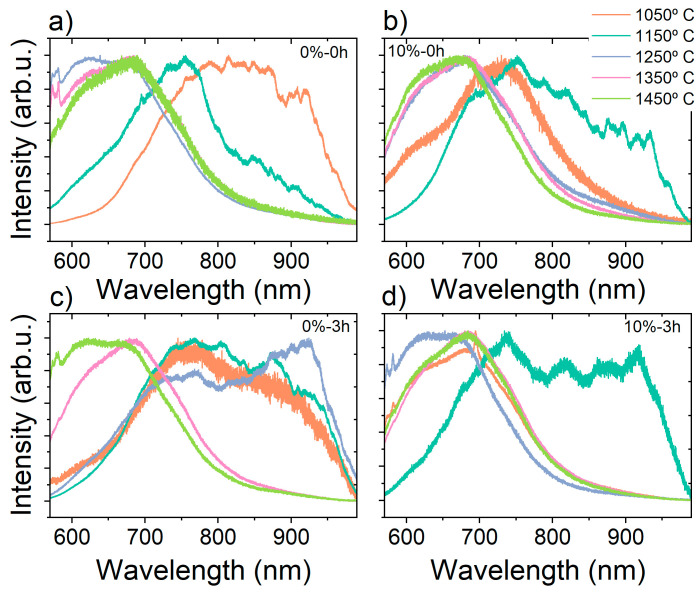
PL measurements for (**a**) 0%-0 h, (**b**) 10%-0 h, (**c**) 0%-3 h, and (**d**) 10%-3 h samples.

These trends were further supported by PL measurements in the visible range (Figure 2). In SiC, carbon antisite vacancies (CAV) are commonly observed near 660 nm, while silicon vacancies appear in the near-infrared region (approximately 858–917 nm for 4H-SiC, for example), depending on polytype and lattice site [6,29].

In the present samples, luminescence peaks near the CAV and V_Si_^−^ emissions are most pronounced at lower reaction temperatures. Notably, all samples synthesized at 1150 °C displayed PL features at approximately 700 nm and/or 900 nm, regardless of Al addition or milling, indicating a dominant temperature effect. Milling further modulated this behavior, as evidenced by differences observed in the 0%-3 h sample set. Aluminum did not appear to facilitate vacancy formation in this system.

**Figure 3:**
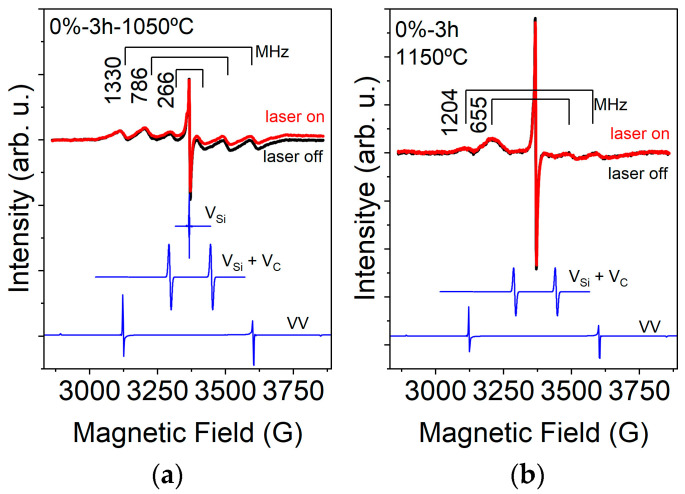
Room-temperature EPR spectra of (**a**) the 0%-3 h-1050 °C sample and (**b**) the 0%-3 h-1150 °C sample, both of which display the additional high-spin features discussed in the text, in addition to the central S = ½ resonance, together with the corresponding EasySpin simulation (version 6.0.12) of the divacancy (D = 1.33 GHz, E = 0) and V_Si_^−^ + CSi (D = 270 MHz) and V_Si_^−^ powder patterns [37].

The same trend was observed in the EPR measurements as well (Figure 3 and Figure S3). Most of the samples showed a typical SiC EPR spectrum, with the main signal attributed to the S = ½ V_C_^+^ and CAV vacancies. It should be noted, however, that in nanocrystalline SiC powders, EPR line assignment is intrinsically less direct than in bulk single crystals because the measured spectrum represents an orientational average over all crystallite axes, while additional structural disorder introduces substantial inhomogeneous broadening, which is relevant especially for paramagnetic defects with higher spin states. For vacancy-related centers with S = 1, only defects retaining predominantly axial symmetry can produce discernible powder singularities, in most cases, associated with the principal orientations $B_{0}∥c$ and $B_{0}⊥c$; by contrast, rhombic or lower-symmetry centers redistribute the spectral intensity over the full anisotropy of the zero-field-splitting (ZFS) tensor and therefore tend to appear as broad, weak, or unresolved features. For S = 3/2 Kramers defects, the persistence of Kramers doublets supports detectability, but the spectral envelope and apparent splitting remain strongly dependent on the ratio of zero-field splitting to the Zeeman energy, making direct comparison with single-crystal derived parameters not straightforward. In the presented SiC nanocrystals, this difficulty is further amplified by surface-induced symmetry breaking, polytype intergrowth, stacking faults, and nanoscale strain, all of which generate distributions in local crystal field, (g)-tensor, and zero-field splitting. Accordingly, the observed powder features should be regarded as broadened superpositions from closely related defect environments, and the assignments proposed below represent the most physically consistent interpretation of the available data.

Nevertheless, the shoulder observed in most samples synthesized below 1250 °C is attributable to the presence of V_Si_^−^ [17,25]. The EPR signal of V_Si_^−^ can have multiple zero-field splitting values near the central signal, depending on the structure and the neighbors around the vacancy. This agrees with the PL results, which showed peaks around the known emission ranges of the V_Si_^−^ and CAV signals for most of our samples synthesized below 1250 °C.

Samples synthesized without Al and with 3 h HEBM at 1050 and 1150 °C exhibited additional signals attributable to other high-spin defects with non-zero ZFS values. The first set of peaks, with D ≈ 1.3 GHz, is present in both the 1050 and 1150 °C samples and is consistent with VV type defects, whose axial configurations in 4H-SiC (hh, kk, C_3V_ symmetry) and 6H-SiC (hh, k_1_k_1_, k_2_k_2_, C_3V_ symmetry) span D values of approximately 1.30–1.36 GHz [5,30,31]. The axial configurations produce the dominant powder features, while the basal configurations (C_1h_ symmetry) contribute only to the diffuse background, consistent with the observed spectral shape.

The second set of peaks, with D ≈ 270 MHz, is observed only in the 1050 °C sample and is absent at 1150 °C. This signal falls numerically close to the D value reported for the EI4 center in 6H-SiC (D ≈ 262–295 MHz) [9,32]; however, EI4 has C_1h_ symmetry and would not produce resolvable powder features, as argued above. A more physically consistent assignment is a negatively charged silicon vacancy modified by an axially placed carbon antisite at approximately 5 Å along the c-axis V_Si_^−^ + C_Si_, for which C_3V_ symmetry is preserved, and the weakened long-range interaction with the distant antisite substantially lowers the ZFS relative to the nearest-neighbor configuration, EI4 [33]. High-throughput first-principles calculations within the ADAQ framework have identified this class of modified silicon vacancy as the only intrinsic double-defect candidate in 4H-SiC, consistent with an S = 3/2 ground state, C_3V_ symmetry, and a ZFS approximately one order of magnitude larger than that of the isolated silicon vacancy, placing D in the several-hundred-MHz range [33]. Its formation is thermodynamically favored under the carbon-excess, silicon-deficient conditions imposed by PTFE decomposition at low synthesis temperature. Critically, the C_Si_–V_C_ pair is known to anneal out rapidly above ~1000–1200 °C in single-crystal 4H-SiC [6,34], which is consistent with the disappearance of the D ≈ 270 MHz signal at 1150 °C in our nanocrystalline samples.

The third prominent signal, with D ≈ 650–780 MHz, is present in both the 1050 and 1150 °C samples. We tentatively assign this signal to a second-nearest-neighbor (2NN) V_Si_–V_C_ pair, in which the increased vacancy-pair separation relative to the nearest-neighbor divacancy (VV) reduces the zero-field splitting via two complementary mechanisms: a purely geometric dipolar term D_dip_ ∝ r^−3^, which depends only on the spin–center separation and is polytype-independent, and a spin–orbit term D_SO_ ∝ λ^2^/Δ_cf_, where λ is the atomic spin–orbit coupling constant and Δ_cf_ is the crystal-field splitting at the vacancy site. For a second-nearest-neighbor geometry, the increased spin-center separation reduces D_dip_ by approximately a factor of two relative to the nearest-neighbor divacancy, consistent with the observed D ≈ 650–780 MHz compared to D ≈ 1.30–1.36 GHz for VV. Axial 2NN VV configurations retain C_3V_ symmetry (in contrast with the V_Si_^−^ + C_Si_ 2NN system in which the vicinity of C_Si_ reduced the C_T_ symmetry of V_Si_^−^ affecting D_SO_), ensuring E ≈ 0 and a resolvable axial powder pattern consistent with the features detected here. An alternative assignment, a V_Si_^−^ + C_Si_ complex at second-nearest-neighbor distance in 4H-SiC, analogous to the distant V_Si_^−^ + C_Si_ proposed for the 270 MHz signal, which, at least predicted theoretically [33], cannot be entirely excluded on structural grounds. However, the observation that the D ≈ 270 MHz signal disappears between 1050 and 1150 °C while the D ≈ 650–780 MHz signal persists at both temperatures argues strongly against a common C_Si_-based origin for the two signals. The 4H/6H ratio is similar between the two samples, ruling out a polytype-driven change in ZFS as the explanation. Instead, the differential thermal behavior reflects the distinct stabilities of the underlying defect families: the C_Si_–V_C_ complex anneals out at ~1000–1200 °C, while the divacancy and divacancy-like vacancy-pair centers are stable well above 1400 °C [6,35,36], consistent with the persistence of the D ≈ 650–780 MHz signal at 1150 °C and its assignment to a VV-derived rather than C_Si_^−^-derived configuration

Upon illumination, the EPR spectrum exhibited a subtle but reproducible change, manifesting predominantly as a modification of the spectral background rather than a pronounced variation in the intensities of the well-resolved peaks. This behavior is consistent with the photo-induced generation—or charge-state conversion—of paramagnetic defect centers characterized by large and/or broadly distributed ZFS parameters, whose powder-pattern envelope spans a magnetic-field range far exceeding the observation window, rendering them effectively indistinguishable from the baseline.

**Figure 4:**
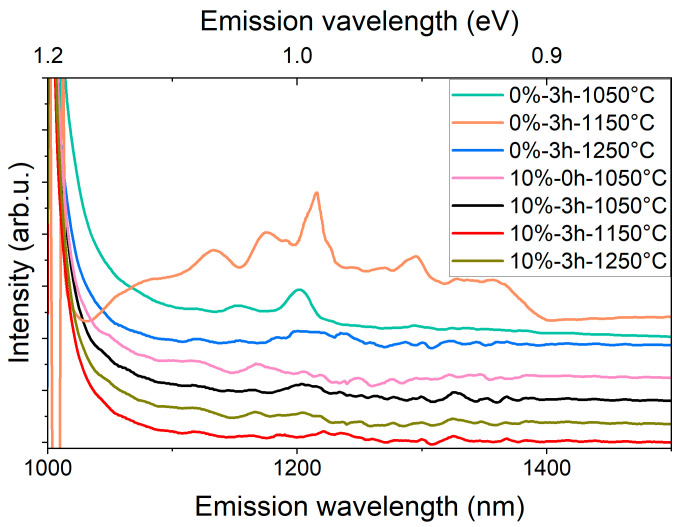
Room-temperature near-infrared PL spectra (980 nm excitation) of selected samples chosen after the EPR analysis. The low-temperature samples (1050–1150 °C) show characteristic NIR PL features attributable to divacancy-related color centers, whereas the high-temperature reference samples exhibit no detectable PL signal above the detector dark-count level under identical acquisition conditions.

We therefore also probed the photoluminescence properties in the infrared range. Consistent with the EPR results, only samples synthesized at 1050 °C and 1150 °C exhibited significant PL signals in the infrared range (Figure 4).

The most prominent PL signal was recorded from the 0%-0 h-1050 °C sample, which also showed changes in EPR spectra under illumination. The broad PL signal also contains several narrower features. Even though the measurement conditions do not allow detailed evaluation of the signal, many of the narrower bands have maxima close to the known ZPL lines of infrared defects in SiC (Table 1).

It should be noted that the sample with the highest concentration of high-spin defects is the one that is poorly hexagonal and was formed at a relatively low synthesis temperature. This suggests a reaction mechanism that differs from the general synthesis route. Due to the presence of PTFE in the precursor mixture, the reaction can proceed through a pathway described by the following reactions [21]:

n Si_(s)_ + (-CF_2_CF_2_)_n(g)_ → n SiF_4(g)_ + 2n C_(s)_ (1)

Si_(s,l)_ + C_(s)_ → SiC_(s)_ (2)

The first reaction is exothermic and can locally melt Si, enabling reaction with carbon to form SiC. During this process, fluorine reacts with silicon to form the volatile SiF_4_, leading to silicon depletion in the system [21,43]. This silicon deficiency explains the PL and EPR results, particularly the presence of silicon-vacancy-related emissions in samples synthesized at lower temperatures. The decomposition of PTFE also increases the carbon content, altering the initial C/Si ratio and creating excess carbon that can act as a diluent, thereby lowering the combustion temperature, consistent with previous observations [21].

Defective structures are common in samples synthesized at lower temperatures. Shorter high-temperature dwell times limit sustained Si melting and reaction completion, leading to lower yields and higher defect concentrations. Conversely, higher-temperature samples allow the second reaction step to proceed for longer, producing more stable, crystalline SiC. Product yield showed that HEBM erases the active sites on the reactant powders, increasing the ignition temperature. This behavior is consistent with PTFE-induced initiation of a low-temperature chemical reaction that forms some hexagonal SiC below the ignition temperature of the elemental Si–C system.

When Al is introduced, it competes with Si for reaction with PTFE according to [20]:

4 Al_(s)_ + 3 (-CF_2_CF_2_)_n(g)_ → 4n AlF_3(s)_ + 6n C_(s)_ (3)

This competition reduces the likelihood of silicon vacancy formation, as PTFE can react with either Si or Al. The suppression of silicon vacancies in Al-containing samples is confirmed by both PL and EPR data.

Ball milling further modifies the reaction pathway. Milling brittle materials such as Si and C leads to repeated particle fracture and size reduction [44]. However, when a ductile metal such as Al is present, milling behavior changes significantly. Al particles plastically deform, flatten, and adhere to brittle particles, temporarily shielding them from fracture [45]. Over time, these Al layers wear off, but the cold-welding process introduces additional defects that enhance diffusion during subsequent synthesis, influencing crystallite size and reaction kinetics [46].

## 4. Conclusions

This work demonstrates that temperature and HEBM are the primary factors controlling SiC formation, polytype distribution, and defect generation in Si–PTFE–C based synthesis under oxygen-free conditions. We show that a chemical reaction is possible below the ignition point of the Si-C system. Low reaction temperatures and milling favor the formation of point defects, particularly silicon vacancies and divacancies.

Defect characterization by PL and EPR consistently shows that lower synthesis temperatures and milling conditions maximize defect concentrations, whereas higher temperatures yield more stable, less defective SiC. A critical temperature of ~1050 °C after 3 h of HEBM produces characteristic defect-related PL and EPR features in Al-free samples. Aluminum does not promote defect formation; instead, it suppresses silicon vacancies by competing with Si for reaction with PTFE, as confirmed by both PL and EPR. The formation of hexagonal SiC without detectable cubic content opens the way for fine control of reaction time and heating profiles, decoupling temperature effects from total thermal exposure and enabling better understanding of defect stabilization mechanisms. Exploring controlled Al concentrations or alternative metal additives may allow more precise tuning of defect populations without suppressing desired vacancies.

A more detailed identification of the EPR and PL centers is possible after the aforementioned optimization and engineered post-annealing procedures. Temperature-dependent EPR and optically detected measurements could further support the identification of the EPR signals, as D_SO_ and D_dip_ exhibit different temperature dependencies.

## Figures and Tables

**Table 1:** Information about different possible defects, such as ZPL, D, and E values for different SiC polytypes.

Defect	Polytype	ZPL	D (E) Values	Ref
VV (neutral divacancy)	3C-SiC	~1130 nm	~1330 MHz	[7]
VV (PL1–PL4)	4H-SiC	PL1: 1132.0 nm; PL2: 1130.5 nm; PL3: 1107.6 nm; PL4: 1078.5 nm	~1300–1360 MHz	[38]
VV (QL1–QL6)	6H-SiC	QL1: 1139.6 nm; QL2: 1135.0 nm; QL3: 1123.9 nm; QL4: 1107.4 nm; QL5: 1093.5 nm; QL6: 1093.0 nm	~1140–1360 MHz	[30]
PL6	4H-SiC	~1038–1040 nm	~1365 MHz	[31]
PL7	4H-SiC	~1106 nm	~1333 MHz	[39]
NV^−^	3C-SiC	~1289 nm	-	[40]
NV^−^	4H-SiC	1241, 1242, 1223, and 1180 nm	~1300 MHz	[41]
NV^−^	6H-SiC	1240.9, 1226.4, 1203.1, 1182.9, 1182.6, 1153.7 nm	~1300 MHz	[42]
EI4	4H-SiC	-	~1030 MHz (~195 MHz)	[34]
EI4	6H-SiC	-	262—295 MHz	[9]

## Data Availability

The original contributions presented in this study are included in the article/Supplementary Materials. Further inquiries can be directed to the corresponding authors.

## Supplementary Materials

The following supporting information can be downloaded at: https://www.mdpi.com/article/10.3390/nano16100627/s1, Figure S1. PXRD diffractograms measured for all samples. Table S1. Selected peak positions of 3C-SiC and their indices. Table S2. Selected peak positions of 2H-SiC and their indices. Table S3. Selected peak positions of 4H-SiC and their indices. Table S4. Selected peak positions of 6H-SiC and their indices. Table S5. Selected peak positions of Si and their indices. Table S6. Selected peak positions of Al_2_O_3_ and their indices. Figure S2. Raman spectra were measured for all samples. Figure S3. EPR measurements for all the samples. Table S7. Quantitative Raman parameters extracted from Figure S2 for the Al-free, 3 h HEBM samples.
