# Supplementary material for: Controlled Chemical Synthesis of Color Centers in Nanocrystalline Silicon Carbide
Source: Nanomaterials (Basel). 2026 May 19;16(10):627. doi: 10.3390/nano16100627 (PMC13209189; doi:10.3390/nano16100627)
Supplement: Supplementary file 1 [file nanomaterials-16-00627-s001.zip › nanomaterials-4327447-supplementary.pdf]

nanomaterials

MDPI

Supplementary Materials

Controlled Chemical Synthesis of Color Centers in Nanocrystalline Silicon Carbide

Sarah Morais Bezerra [1,2], Gabor Bortel [1], Sandor Kollarics [1,3,4,5], Adam Gali [1,6,7] and David Beke [1,8].

1 HUN-REN Wigner Research Centre for Physics, Institute for Solid State Physics and Optics, H-1121 Budapest, Hungary
2 Department of Physical Chemistry and Materials Science, Faculty of Chemical Technology and Biotechnology, Budapest University of Technology and Economics, Műegyetem Rakpart 3, H-1111 Budapest, Hungary
3 SOLEIL Synchrotron, L'Orme des Merisiers, RD128, 91190 Saint Aubin, France
4 Department of Physics, Institute of Physics, Budapest University of Technology and Economics, Müegyetem Rakpart 3, H-1111 Budapest, Hungary
5 HUN-REN–BME Condensed Matter Research Group, Budapest University of Technology and Economics, Müegyetem Rakpart 3, H-1111 Budapest, Hungary
6 Department of Atomic Physics, Institute of Physics, Budapest University of Technology and Economics, Müegyetem Rakpart 3, H-1111 Budapest, Hungary
7 MTA-WFK Lendület "Momentum" Semiconductor Nanostructures Research Group, H-1525 Budapest, Hungary
8 Kandó Kálmán Faculty of Electrical Engineering, Óbuda University, Tavaszmező 17, H-1084 Budapest, Hungary

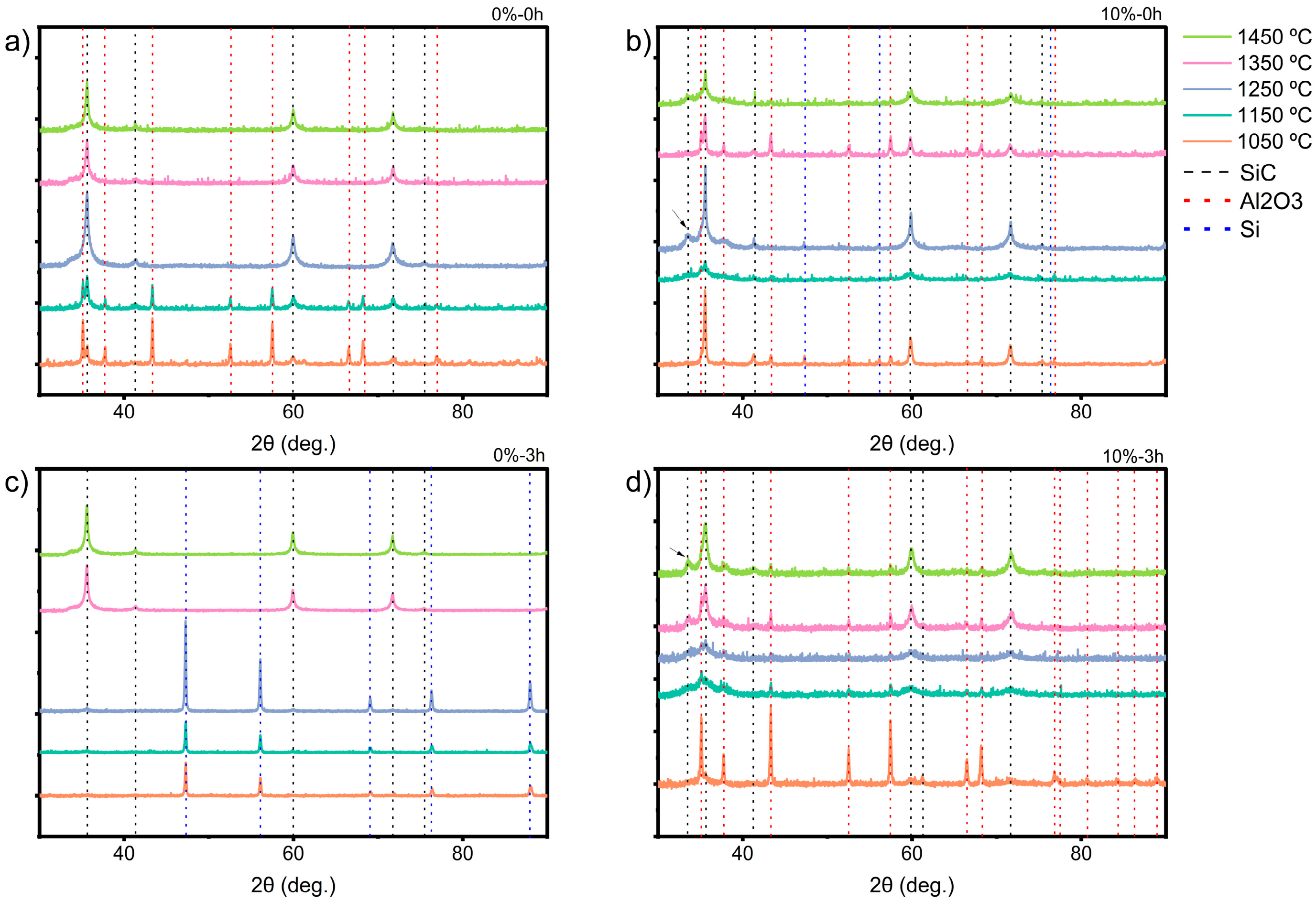

Figure S1. PXRD diffractograms measured for all samples. Arrows indicate the Warren-type asymmetric features in the 33–35° 2θ region of the (00ℓ) reflections of the hexagonal polytypes, which are attributable to stacking-fault density.

Table S1. Selected peak positions of 3C-SiC and their indices.

Indices (hkl)	2θ (degrees)
111	35.65
200	41.40
220	59.99
222	75.52
311	71.78
400	89.99

Table S2. Selected peak positions of 2H-SiC and their indices.

Indices (hkl)	2θ (degrees)
002	35.23
004	74.50
100	33.30
101	37.81
102	49.26
103	64.94
104	84.08
$2\bar{1}0$	59.52

$2\bar{1}2$	71.09
200	69.94
201	72.71
202	80.80
203	93.96
$3\bar{1}0$	98.61

Table S3. Selected peak positions of 4H-SiC and their indices.

Indices (hkl or hkil)	2θ (degrees)
002	17.63
004	35.70
006	54.75
008	75.63
0010	100.07
100	33.97
101	35.15
102	38.52
103	43.65
104	50.10
105	57.61
106	66.02
107	75.31
108	85.56
109	97.03
$2\bar{1}0$	60.79
$2\bar{1}2$	63.83
$2\bar{1}4$	72.54
$2\bar{1}6$	86.27
200	71.50
201	72.21
202	74.32
203	77.79
204	82.57
205	88.65
206	96.07

Table S4. Selected peak positions of 6H-SiC and their indices.

Indices (hkl or hkil)	2θ (degrees)
002	11.71
004	23.54
006	35.64
008	48.17
0010	61.34
0012	75.49
0014	91.15
100	33.58
101	34.11
102	35.68
103	38.16
104	41.42
105	45.33
106	49.77

107	54.68
108	59.99
109	65.69
1010	71.78
1011	78.26
1012	85.20
1013	92.66
2$\bar{1}$0	60.04
2$\bar{1}$2	61.41
2$\bar{1}$4	65.41
2$\bar{1}$6	71.82
2$\bar{1}$8	80.43
2$\bar{1}$10	91.21
200	70.58
201	70.90
202	71.84
203	73.40
204	75.57
205	78.32
206	81.65
207	85.56
208	90.03
209	95.11
3$\bar{1}$0	99.68
3$\bar{1}$1	99.99

Table S5. Selected peak positions of Si and their indices.

Indices (hkl)	2θ (degrees)
111	28.46
220	47.34
222	58.90
311	56.17
331	76.44
333	95.05
400	69.19
422	88.11
511	95.05

Table S6. Selected peak positions of Al_2O_3 and their indices.

Indices (hkl or hkil)	2θ (degrees)
006	41.70
0012	90.78
10$\bar{8}$	61.34
10$\bar{2}$	25.59
104	35.17
1010	76.93
2$\bar{1}$0	37.79
2$\bar{1}$3	43.37
2$\bar{1}$6	57.53
2$\bar{1}$9	77.29
20$\bar{1}$0	89.06
20$\bar{4}$	52.58

202	46.20
208	74.35
$3\bar{1}\bar{8}$	86.56
$3\bar{1}\bar{5}$	70.46
$3\bar{1}\bar{2}$	61.16
$3\bar{1}1$	59.77
$3\bar{1}4$	66.56
$3\bar{1}7$	80.47
$30\bar{6}$	83.27
300	68.25
306	83.27
$4\bar{2}0$	80.75
$4\bar{2}3$	84.41
$4\bar{2}6$	95.31
$4\bar{1}\bar{4}$	91.25
$4\bar{1}\bar{1}$	85.19
$4\bar{1}2$	86.41
$4\bar{1}5$	94.88
$40\bar{2}$	98.46

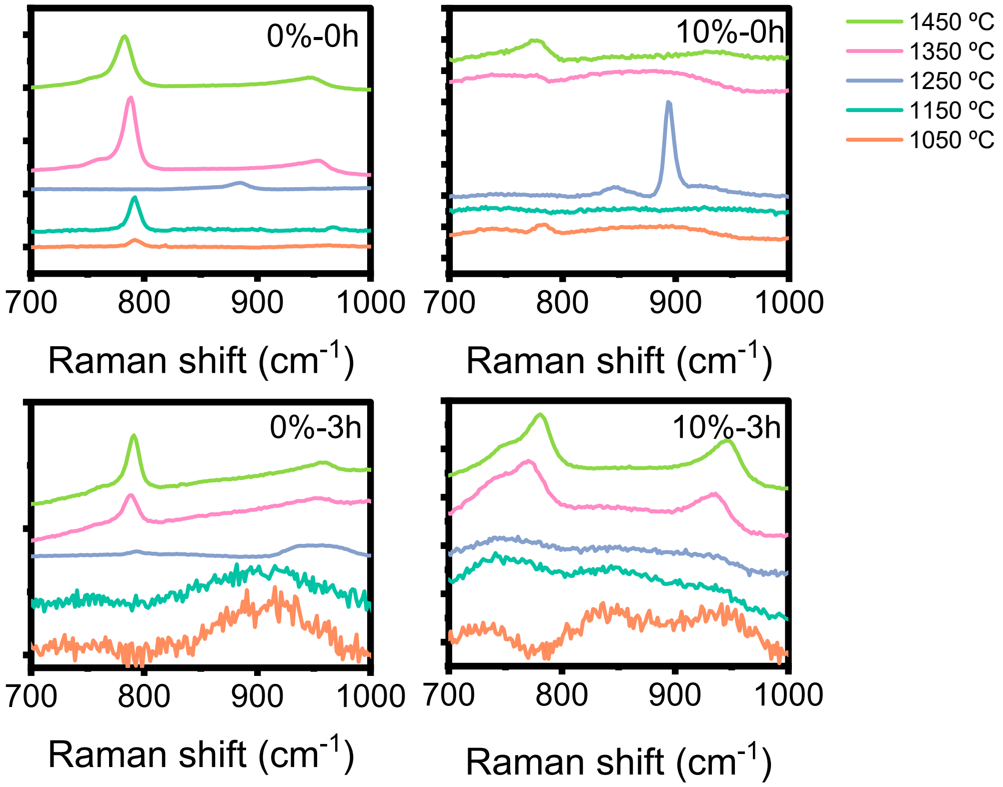

Figure S2. Raman spectra were measured for all samples. Some samples exhibited very high background luminescence, resulting in a low signal-to-noise ratio. The dominant SiC features are the TO mode at ~796 cm^{-1} and the LO mode at ~972 cm^{-1}. A broad disorder-related background is visible between 700 and 950 cm^{-1}. Quantitative parameters are given in Table S7.

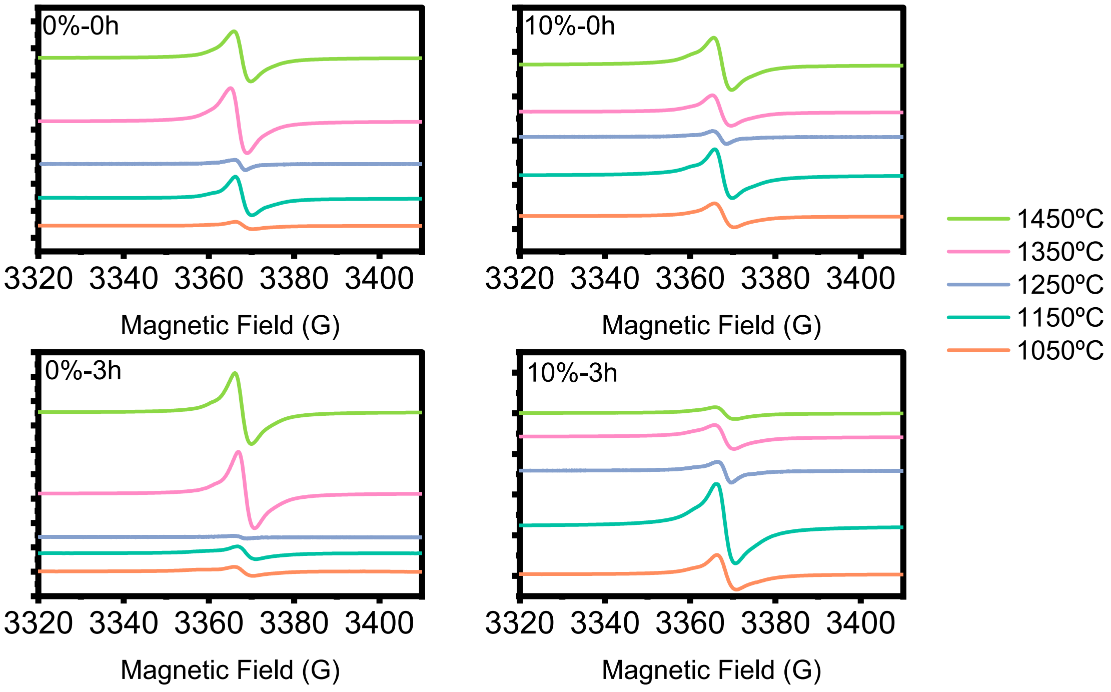

Figure S3. EPR measurements for all the samples.

Table S7. Quantitative Raman parameters extracted from Figure S2 for the Al-free, 3 h HEBM samples: full width at half maximum (FWHM) of the SiC TO mode (~796 cm^{-1}), the integrated intensity ratio $I_{disorder}/I_{TO}$ of the 700–950 cm^{-1} disorder background to the TO mode, and the relative amorphous-carbon ratio I_D/I_{TO}. All values are approximate and intended as relative indicators of structural disorder.

**Sample**	**TO FWHM (cm^{-1})**	**TO intensity**
0%–0 h–1050 °C	~12	12 163
0%–0 h–1150 °C	~11.5	62 493
0%–0 h–1250 °C	~6	12 497
0%–0 h–1350 °C	~12	13 5013
0%–0 h–1450 °C	~14	93 402
10%–0 h–1050 °C	13	2 714
10%–0 h–1150 °C	13	1 538
10%–0 h–1250 °C	10	30 196
10%–0 h–1350 °C	25	65 040
10%–0 h–1450 °C	22	7 936
0%–3 h–1050 °C	88	230
0%–3 h–1150 °C	65	250
0%–3 h–1250 °C	14	12 535
0%–3 h–1350 °C	12	20 853
0%–3 h–1450 °C	10	39 980
10%–3 h–1050 °C	60	323
10%–3 h–1150 °C	52	810

10%–3 h–1250 °C	43	6 050
10%–3 h–1350 °C	30	48 028
10%–0 h–1450 °C	19	54 500
